# Bayesian divergence-based approach for genomic multitrait ordinal selection

**DOI:** 10.1093/g3journal/jkaf183

**Published:** 2025-09-02

**Authors:** Bartolo de J Villar-Hernández, Pawan Singh, Nerida Lozano-Ramírez, Paolo Vitale, Guillermo Gerard, Flavio Breseghello, Susanne Dreisigacker, José Crossa

**Affiliations:** International Maize and Wheat Improvement Center (CIMMYT), Km 45, Carretera México-Veracruz, Estado de México CP 52640, México; International Maize and Wheat Improvement Center (CIMMYT), Km 45, Carretera México-Veracruz, Estado de México CP 52640, México; International Maize and Wheat Improvement Center (CIMMYT), Km 45, Carretera México-Veracruz, Estado de México CP 52640, México; International Maize and Wheat Improvement Center (CIMMYT), Km 45, Carretera México-Veracruz, Estado de México CP 52640, México; International Maize and Wheat Improvement Center (CIMMYT), Km 45, Carretera México-Veracruz, Estado de México CP 52640, México; International Maize and Wheat Improvement Center (CIMMYT), Km 45, Carretera México-Veracruz, Estado de México CP 52640, México; International Maize and Wheat Improvement Center (CIMMYT), Km 45, Carretera México-Veracruz, Estado de México CP 52640, México; International Maize and Wheat Improvement Center (CIMMYT), Km 45, Carretera México-Veracruz, Estado de México CP 52640, México; Colegio de Postgraduados, Montecillos, Estado de México CP 56230, México

**Keywords:** Bayesian genomic selection, ordinal traits, Kullback–Leibler divergence, Bhattacharyya distance, Hellinger distance, decision theory, parental selection, wheat breeding, MPS-R package, genomic prediction

## Abstract

Effective genomic selection for ordinal traits, such as disease resistance scores, is a persistent challenge in plant breeding due to the discrete, ordered nature of these phenotypes. This study presents a novel Bayesian divergence-based framework for multitrait ordinal selection, implemented in the extended Multitrait Parental Selection R package (MPS-R). By leveraging decision-theoretic loss functions, including the Kullback–Leibler (KL) divergence, Bhattacharyya distance, and Hellinger distance, our approach quantifies the distance between candidate distributions and breeder-defined target distributions. Through extensive simulations under 6 scenarios combining different genetic correlation structures and heritability levels, we demonstrate the comparative performance of each loss function. KL divergence consistently yielded superior genetic gains, especially in moderate heritability settings. Additionally, random sampling validation using real wheat disease resistance data confirmed the utility of these methods in practical breeding contexts. The MPS-R package implements this methodology through user-friendly functions tailored for ordinal trait selection in breeding applications. Our results demonstrate that this toolset provides a flexible, robust, and biologically grounded framework to enhance selection efficiency in breeding programs targeting complex, multitrait ordinal phenotypes. A couple of limitations employed by the simulation scheme used on the study are also discussed.

## Introduction

Categorical and ordinal traits are fundamental to plant breeding programs, particularly for evaluating disease resistance, stress tolerance, and other key agronomic characteristics. These traits are typically recorded on discrete, ordered scales, for example, from “resistant” to “highly susceptible,” and often reflect complex polygenic responses to environmental pressures (Gebhardt and von Bothmer 2019; Bhat et al. 2021). However, their discrete and nonequidistant nature poses significant challenges to conventional genomic selection (GS) methods, which generally assume continuous, normally distributed traits with interval-scale measurement properties. Continuous traits are measured on an infinite scale that supports arithmetic operations and meaningful differences between values. In contrast, ordinal traits are composed of discrete, ordered categories where the spacing between levels is not necessarily uniform or interpretable. For example, disease resistance can be classified into 4 levels and coded as follows: resistant = 0, moderately resistant = 1, moderately susceptible = 2, and highly susceptible = 3. However, the difference in levels, such as the difference between 0 and 1 vs 2 and 3, does not reflect consistent or quantifiable changes in underlying biology. Furthermore, the numeric encoding of ordinal categories is arbitrary. The same categories could be encoded in reverse order without altering their ordinal relationships. For example, resistant could be coded as 3, moderately resistant as 2, moderately susceptible as 1, and highly susceptible as 0.

Many critical breeding objectives, including resistance to *Fusarium* head blight, rust, and *Septoria tritici* blotch, are commonly evaluated using ordinal scales that quantify increasing severity or resistance. Although these traits are inherently categorical with ordered levels, the intervals between scores are frequently undefined or nonuniform. Despite this, they are routinely treated as continuous variables in genomic prediction and association analyses. This simplification can introduce critical issues. First, ignoring the ordinal nature of the data leads to biased estimation of genetic parameters and suboptimal predictive performance (Montesinos-López et al. 2015). Furthermore, treating ordinal traits as continuous deviates the fundamental assumptions of linear models, including normality and homoscedasticity of errors. This misrepresentation can result in biased inferences and a loss of statistical power. It also obscures the interpretation of genetic effects because the differences between adjacent ordinal categories are not necessarily equal or meaningful in a linear sense. Consequently, effect sizes may be distorted, and genetic signals may be diluted or inflated depending on the distribution of observations across categories. This misrepresentation can further bias estimates of heritability and reduce the efficiency of selection decisions. Finally, imposing a continuous structure on ordinal phenotypes can misrepresent the true genotype–phenotype architecture, potentially masking underlying biological mechanisms and reducing the genetic gain achievable through selection.

Furthermore, many ordinal phenotypes used in plant breeding are not inherently categorical; rather, they represent coarse measurements of underlying, unobserved biological processes. These processes are usually continuous, such as the progression of pathogen colonization or physiological maturity. They evolve on a latent scale that cannot be directly measured with precision. Instead, breeders and researchers often rely on categorical scoring systems that divide this latent continuum into ordered classes for practical reasons, such as feasibility, cost-efficiency, or limitations in phenotyping technology. Consequently, the observed ordinal phenotype is best understood as the result of thresholding a continuous latent variable that reflects the true biological state. Statistical models that acknowledge this latent structure assume that an individual's observed level arises when their unobserved continuous liability crosses certain unknown thresholds. This modeling approach preserves the ordinal nature of the data and accommodates unequal and biologically nonlinear distances between categories. Failing to acknowledge this latent structure and treating ordinal outcomes as nominal or continuous can result in biased estimates of genetic effects, loss of predictive accuracy, and misinterpretation of trait architecture. In genomic prediction, for instance, forcing a continuous structure onto inherently ordinal traits can distort the genotype–phenotype relationship and decrease selection efficiency. This highlights the importance of respecting the measurement scale and the underlying data-generating process in genomic analyses.

To improve the statistical modeling of these traits, Bayesian threshold models have been introduced. These models assume the presence of a latent, unobserved continuous variable that, when segmented by threshold parameters, yields the observed ordinal phenotype (Agresti 2010). Within a Bayesian framework, such models allow for flexible prior specification and full posterior inference, providing both point estimates and uncertainty quantification (Pérez-Rodríguez and de los Campos 2014). While these models enhance the estimation and prediction of ordinal outcomes, they do not inherently incorporate breeders' decision-making processes, specifically the ability to select genotypes based on how closely their predicted phenotypic distributions align with predefined breeding objectives.

Bayesian decision theory is a powerful and flexible framework that allows breeders to advance beyond pure prediction in GS, especially when breeding objectives involve complex trait distributions. Rather than relying solely on point estimates, this framework enables breeders to define a desired outcome as a target distribution and select genotypes whose posterior predictive distributions align most closely with the desired outcome. For instance, an ideal population for a long-term breeding program could be defined as having 70% resistant individuals, 20% moderately resistant individuals, and 10% susceptible individuals. Bayesian predictive models naturally support this paradigm by providing the full posterior predictive distribution for each genotype in the candidate set. This allows breeders to evaluate selection candidates based not only on their expected value but also on how closely their posterior predicted outcome distributions align with the target distribution. Divergence metrics, such as the Kullback–Leibler (KL) divergence, offer a principled way to quantify the distance between these distributions. This makes the selection process both statistically rigorous and decision-oriented. Previous implementations of this approach have demonstrated excellent performance with continuous traits (Villar-Hernández et al. 2018, 2024). However, its application to ordinal traits, which are prevalent in breeding programs, remains largely unexplored. In this study, we propose an extension of the decision-theoretic framework for multitrait ordinal selection. This extension enables more rational and interpretable decision-making that reflects the categorical nature of traits and population-level breeding objectives.

In order to address the current gap in GS methodologies for multiple ordinal traits, we propose a new approach based on Bayesian decision theory. Although quantifying distances between probability distributions is well established in diverse fields and applications (Grimit et al. 2006; Gneiting and Raftery 2007; Komunjer and Owyang 2012), this concept has not been widely adopted in GS. In this study, we implement our methodology in the updated Multitrait Parental Selection (MPS) R package, which supports single- and multitrait ordinal selection. The package integrates 3 divergence metrics, KL divergence, Bhattacharyya distance (BD), and Hellinger distance, to quantify alignment between posterior predictive distributions and breeder-defined target distributions. We evaluated the performance of these metrics through comprehensive simulation studies under 6 distinct scenarios that combined varying levels of heritability and genetic correlation. We also validated the metrics using real wheat disease resistance data. Results consistently demonstrate that KL provides reliable and consistent improvements across various conditions, though the differences among the metrics are generally modest. Our goal is to provide breeders with a tool that is statistically rigorous and practically accessible, supported by intuitive conceptual foundations, real-world examples, and user-friendly software. By explicitly incorporating breeder-defined goals into the selection process, this framework represents a significant advancement in the selection of complex ordinal traits in modern genomic breeding programs.

## Materials and methods

### Key concepts: objective or target distribution, posterior predictive distribution, loss function, and expected posterior loss

Prior to formal definitions, we briefly outline key concepts underlying the Bayesian divergence-based framework for multitrait ordinal selection.

#### Objective or target distribution

A breeding target distribution is a breeder's ideal trait ratio for future crops, like wanting 10% low, 20% medium, and 70% high disease resistance. These probabilities (0.1, 0.2, 0.7) guide selection, plants matching this “recipe” are chosen. It turns breeding goals into clear math, helping pick top candidates efficiently.

#### Posterior predictive distribution

The posterior predictive distribution is the model's forecast of future trait probabilities, combining observed data with prior knowledge. For ordinal traits, it gives the chance a candidate will express each category (e.g. 20% low, 50% medium, 30% high resistance). These probabilities come from Bayesian analysis, blending uncertainty from both data and model parameters. Breeders use these distributions to predict how offspring will perform, selecting those whose probability profiles best match desired breeding targets.

#### Loss functions

Within the framework of Bayesian decision theory, a loss function serves to quantify the economic or biological penalty incurred when suboptimal decisions are made. In the context of genotype selection, these functions evaluate the discrepancy between a candidate's posterior predictive distribution and a predefined breeding target representing the desired phenotypic optimum.

In our approach, loss functions are employed to assess the divergence or distances between each genotype's posterior predictive distribution and an idealized target distribution encoding the breeder's selection objectives. Among the most widely adopted loss functions in this domain are Kullback–Leibler divergence or simply KL (Kullback and Leibler 1951), Hellinger distance (Hellinger 1909), and BD (Nielsen et al. 2010). We will formally present these loss functions in the context of GS later. Hereafter, we will use “divergence” and “distance” as synonyms.

#### Expected posterior loss

The expected posterior loss quantifies the mean discrepancy between a candidate's posterior predictive distribution and the target distribution, computed by integrating the loss function (e.g. KL divergence) over the posterior. Each candidate is assigned a scalar value representing its average risk; selection prioritizes individuals minimizing this metric, thereby optimizing alignment with breeding objectives under model uncertainty.

## Formal framework for Bayesian ordinal selection

Suppose in a breeding program the interest is to focus on individuals expressing an ordinal trait with $n_{j}$ levels. The random variable $Y_{i}\in 1,\ldots ,n_{j}$ represents realization of the discrete trait. If we have *p* regressors like molecular markers, $x_{ij}$, then our data takes the form of $\left\{(x_{i},y_{i}),\;i=1,\ldots ,N\right\}$, where $x_{i}=(x_{i1},\ldots ,x_{ip}{)}^{T}\in \;R^{p}$ and $y_{i}\in \{1,2,\ldots ,n_{j}\},\;n_{j}\geq 2$, where each realization of $y_{i}$ represents ordered categories, and the categories are not equidistant from each other. For example, plant vigor could have 3 categories (low =1, medium =2, high =3); in this case, $n_{j}=3$. *N* represents the sample size or number of genotypes.

The probability under trait *t* of observing a particular level *c* for individual *i*th, $p_{ic}=P_{r}(y=c),\;c=1,2,\ldots ,n_{j}$, can be linked to predictors using a nonlinear function f(⋅) that in most cases is the probit function that takes the linear predictor $\eta_{i}=x_{i}^{T}\beta$ as input and a threshold parameter γ∈R associated with an unknown latent variable ℓ∈R. Hereafter, we will use “level” or “category” as synonyms. Mathematically,


$$p_{ic}=\Phi (\eta_{i}-\gamma_{c})-\Phi (\eta_{i}-\gamma_{c-1}),$$

where Φ(⋅) represents the cumulative standard normal distribution. The latent variable or latent trait, ℓ, can be interpreted as follows: rather than observing ℓ directly, we observe its categorical version, which is determined by $y_{i}=c\;\mathrm{if},\;\mathrm{and}\;\mathrm{only}\;\mathrm{if},\;\gamma_{c-1}\leq \ell_{i}\leq \gamma_{c}$.

For an individual, under single trait, all probabilities can be collected in a vector of probabilities of length equal to $n_{j}$, i.e. $p_{i}=(\;p_{i1},\ldots ,p_{in_{j}})$. In the example of plant vigor with 3 ordinal levels ($n_{j}=3$), $p_{i1}$ represents the probability of observing low vigor, $p_{i2}$ represents the probability of observing medium vigor, and $p_{i3}=p_{in_{j}}$ represents the probability of observing high vigor. From basic probability theory, we know that $p_{i}$ represents a probability mass function (pmf) and that the sum of all its elements equals 1.

In words, when modeling ordinal phenotypes, predictions are naturally expressed as probabilities associated with each possible level rather than as single numerical values due to the trait's discrete and ordered structure. Threshold models provide a principled framework for this task by assuming that the observed level arises from an underlying latent continuous variable that is segmented by a set of thresholds. The model then estimates the probability that the latent variable falls within the interval corresponding to each ordinal level. This results in a probability of vector for each genotype.

If each genotype has *t* ordinal traits, the problem expands into a multivariate ordinal regression setting. For each genotype *i*, there are *t* ordinal traits, then $Y_{ij}$ is the random variable representing phenotypes observed in the *j*th ordinal trait for individual *i*. Realizations of trait *j* have $n_{j}$ ordered levels, where $n_{j}>2$. The aim is to predict the probability distributions over all possible categories for each of the *t* ordinal traits. Specifically, for trait *j*, the probability of an individual *i* having a level *c* is given by


$$p_{ic}^{\left(j\right)}=P_{r}(Y_{i}^{\left(j\right)}=c),\;\;\;c\in \{1,2,\ldots ,n_{j}\}.$$

**Fig. 1.**
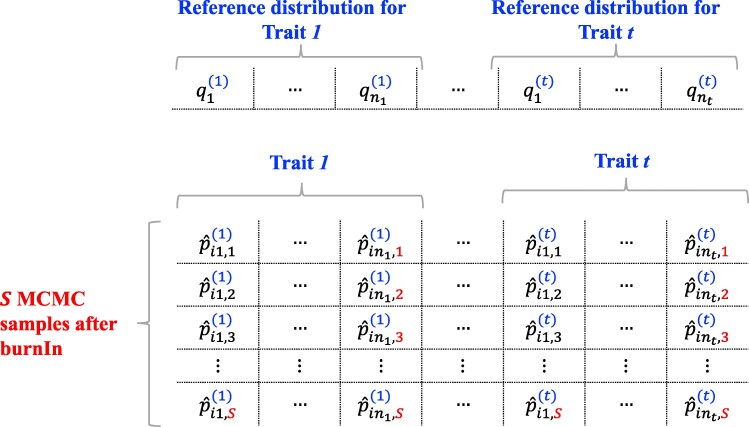
Each trait has a defined reference distribution. For each *i*th line in the candidate set of potential parents, the EL is computed by iterating over all MCMC samples and averaging the results. The best lines are those with the minimum posterior loss.

These probabilities for each genotype *i* can be collected into a vector $p_{i}^{\left(j\right)}=(\;p_{i1}^{\left(j\right)},p_{i2}^{\left(j\right)},\ldots ,p_{in_{j}}^{\left(j\right)})$. A view of vector $p_{i}^{\left(j\right)}$ is depicted in Fig. 1. One row in the figure represents a prediction of $p_{i}^{\left(j\right)}$ for one candidate of selection that involves *j* ordinal traits.

Bayesian regression models used in GS have a clear advantage over traditional approaches. They provide full posterior predictive distributions instead of punctual predictions as in frequentist statistics. Genotypes' posterior predictive distributions are usually in the form of Markov Chain Monte Carlo (MCMC) samples. For example, all models coded in the popular BGLR-R package are Bayesian, and with a little caution, the user can recover all prediction in the form of MCMC objects (Pérez-Rodríguez and de los Campos 2014). This probabilistic output is essential for quantifying uncertainty in predictions and allows breeders to assess their confidence in selecting a particular genotype. This is especially relevant when decisions have long-term consequences in breeding pipelines. Furthermore, Bayesian regression models allow for flexible integration with complex selection goals, such as those involving multitrait or ordinal outcomes, where point predictions may be insufficient or misleading. By propagating parameter uncertainty throughout the model, Bayesian methods yield calibrated predictions that better reflect the variability inherent in biological data.

### Objective distribution

For ordinal traits, breeding candidate predictions are represented as probability vectors corresponding to the pmf of the trait. To identify the best candidates for the next generation, it is essential to define a reference pmf that encapsulates the breeder's selection goals. For example, in a long-term breeding program, a breeder may want a particular trait with 3 categories to be expressed 10% of the time as level 1, 20% of the time as level 2, and 70% of the time as level 3. In this case, the reference distribution is defined as q=(0.1,0.2,0.7). However, another breeder may have slightly different long-term expectations, such as q=(0.05,0.1,0.85), implying a preference for level 1 to appear 5% of the time, level 2 to appear 10% of the time, and level 3 to appear 85% of the time. These examples illustrate that the reference distribution is subjective and shaped by each breeding program's specific goals and priorities. Note that elements of *q* must sum to 1 to represent a valid probability distribution.

From a decision theory perspective, the goal is to select candidates whose probability distributions are as close as possible to this reference, thus ensuring alignment with the breeding objectives. This idea is not entirely new. It was first introduced by Villar-Hernández et al. (2018) for quantitative traits and later implemented in the MPS-R package as described by Villar-Hernández et al. (2024).

In general, breeders aim to optimize multiple ordinal traits simultaneously, and this concept can be extended to multivariate scenarios. Then, suppose a reference distribution $q^{\left(j\right)}=(q_{1}^{\left(j\right)},\;q_{2}^{\left(j\right)},\ldots ,q_{n_{j}}^{\left(j\right)})$ represents breeder's expectation. This distribution plays the role of the truncated distribution in quantitative traits (Villar-Hernández et al. 2018). To select superior individuals, it is necessary to compute the distance of each candidate distribution $p_{i}^{\left(j\right)}$ with respect to $q^{\left(j\right)}$ and select candidates with closest as possible to $q^{\left(j\right)}$. There are some approaches to compute distances, such as the following.

### Three useful loss functions for GS

KL (Kullback and Leibler 1951) divergence is a widely used measure of dissimilarity between probability distributions with applications in several fields. In genomics and plant breeding, its application in GS has shown potential for both single and multiple traits in quantitative traits, offering a promising approach to improve the performance of long-term breeding programs (Villar-Hernández et al. 2018).

To illustrate the computation and interpretation of the KL divergence in GS with ordinal traits, consider a simplified example with a single trait and 3 ordered categories. Suppose the breeder has defined a reference distribution, *q*, representing the long-term selection goals defined as q=(0.7,0.2,0.1), where each component corresponds to the desired proportion of individuals falling into each trait level. Now, imagine that we are evaluating a candidate line and obtain a single realization from its posterior predictive distribution via MCMC, yielding p=(0.3,0.4,0.3). The KL divergence between *p* and *q* quantifies how much the candidate's predicted distribution deviates from the breeder's target. It is calculated as follows:


$$0.3\;*\;\log\;\left(\frac{0.3}{0.7}\right)+0.4\;*\;\log\;\left(\frac{0.4}{0.2}\right)+0.1\;*\;\log\;\left(\frac{0.3}{0.1}\right)=0.35$$

This scalar value summarizes the divergence in terms of information loss if the candidate's predictive distribution *p* is used instead of the ideal *q*. More generally, for any trait with $n_{j}$ ordered categories, the KL divergence between 2 discrete distributions, $p=(\;p_{1},\ldots ,p_{n_{j}})$ and $q=(q_{1},\ldots ,q_{n_{j}})$, is defined as follows:


$$D_{KL}(p||q)=\overset{n_{j}}{\underset{i=1}{\sum }}\;p_{i}\log\;\left(\frac{\;p_{i}}{q_{i}}\right).$$

This measure plays a key role in decision-theoretic selection by guiding breeders toward genotypes whose predicted trait distributions align most closely with their objectives.

When multiple ordinal traits are involved, the generalization of the KL divergence must account for the joint distributions across all traits and is formulated as


$$D_{KL}(p||q{)}_{i}=\overset{t}{\underset{\;j=1}{\sum }}\;\overset{n_{j}}{\underset{i=1}{\sum }}\;p_{i}^{\left(j\right)}\log\;\left(\frac{p_{i}^{\left(j\right)}}{q^{\left(j\right)}}\right),$$

where $p_{i}^{\left(j\right)}$ and $q^{\left(j\right)}$ was described previously and represent the joint distributions across all traits. This formulation allows breeders to evaluate the alignment of a candidate line with multitrait breeding goals by aggregating deviations across all traits. Each trait contributes independently to the overall measure of divergence, enabling breeders to incorporate multiple categorical objectives into the selection process. The multitrait extension preserves the interpretability and flexibility of the decision-theoretic framework while scaling it to address the complexity of modern breeding programs, which must optimize multiple traits simultaneously.

In multitrait ordinal selection, it is important to recognize that the reference distribution may differ for each trait, reflecting the breeder's distinct long-term goals. Consider, for example, a breeding program focused on 2 ordinal traits: disease resistance and plant height. For disease resistance, the breeder might define the target distribution as $q^{\left(1\right)}\;=\;(0.7,\;0.2,\;0.1)$, aiming for 70% resistant individuals, 20% moderately resistant individuals, and 10% susceptible individuals. In contrast, for plant height, with categories such as short, medium, and tall, the breeder might prefer a more balanced outcome, such as $q^{\left(2\right)}=\;(0.3,\;0.4,\;0.3)$. These examples demonstrate how breeders can specify trait-specific reference distributions and how a decision-theoretic framework can accommodate these preferences flexibly and interpretably.

Similarly, other loss measures can be considered. In this paper, we explore 2 additional metrics, one of which is the BD, a widely used loss for comparing probability distributions and assessing target level separability. BD possesses strong statistical properties related to Bayes error and Fisher information (Nielsen et al. 2010). However, its application in GS for plant breeding remains largely unexplored. For our purpose, the BD in multitrait setting is formulated as


$$D_{B}(p,\;q{)}_{i}=\overset{t}{\underset{\;j=1}{\sum }}-\ln\;\left(\overset{n_{j}}{\underset{i=1}{\sum }}\;\sqrt{p_{i}^{\left(j\right)}\cdot q^{\left(j\right)}}\right).$$

The equation shares the same interpretation as the KL divergence: genotypes with the smallest distances to the reference distribution are prioritized for selection.

Finally, we present the Hellinger loss (Hellinger 1909), a metric used to quantify the dissimilarity between probability distributions. The Hellinger distance and the BD are closely related; however, the Hellinger distance is a true metric, whereas the Bhattacharyya coefficient does not satisfy the triangle inequality (Callahan 2019). In multicriteria decision-making, the Hellinger distance has been used to directly compare probability distributions, minimizing potential information loss from data transformations (Lourenzutti and Krohling 2014). In addition, this distance estimator can be reinterpreted as a maximum likelihood estimator with a penalized log-likelihood function, providing insight into its robustness properties (Harris and Ayanendranath 1994). Its mathematical formulation is given by


$$D_{H}(p,q{)}_{i}=\overset{t}{\underset{\;j=1}{\sum }}\frac{1}{\sqrt{2}}\sqrt{\overset{n_{j}}{\underset{i=1}{\sum }}\;\left(\sqrt{p_{i}^{\left(j\right)}}-\sqrt{q^{\left(j\right)}}\right)}.$$

### Bayesian calculation of distances or expected loss

Categorical regression models can be conceptualized within the framework of latent variable models, specifically threshold models. These models can be implemented from both classical (frequentist) and Bayesian perspectives. However, we advocate Bayesian methods due to their inherent advantages, particularly their ability to provide uncertainty estimates for individual predictions throughout posterior distributions. This is particularly valuable in GS, where quantifying the reliability of predictions is critical to decision-making.

Bayesian threshold models are a valuable alternative to conventional linear approaches for predicting ordinal traits genomically, especially since they account for the discrete and ordered nature of the data. Several studies have reported notable improvements in predictive performance when applying threshold models. For example, Song et al. (2024) achieved gains in prediction accuracy ranging from 1.2 to 2.9% for categorical fish traits, and Wang et al. (2013) documented improvements of up to 30.4% for binary traits in simulation settings. Among Bayesian implementations, threshold models are flexible and effective framework for modeling ordinal traits. It frequently outperforms traditional dichotomization strategies. Comparative analyses have shown that threshold models surpasses machine learning techniques, such as support vector machines and neural networks, in predicting ordinal traits in plants (Montesinos-López et al. 2019). As GS continues to evolve, threshold-based methodologies offer a promising approach for achieving higher genetic gains in complex, categorical traits.

Suppose a Bayesian ordinal regression model has been fitted for each trait of interest in a breeding program. After discarding a predetermined number of MCMC samples (burn-in), S stationary samples are obtained from the posterior distributions (Fig. 1). The expected posterior loss is then calculated by evaluating the relevant equations S times and averaging the results,


$$\bar{L}=\frac{\sum_{l=1}^{S}\;D_{l}}{S},$$

where *D* represents any of the 3 loss functions presented in this paper. Top lines are those with the minimum posterior loss.

### Prediction accuracy evaluation

In our study, we evaluated the predictive performance of threshold models using 3 complementary metrics: the Brier score, the ranked probability score, and the proportion of correctly predicted categories. These metrics capture different aspects of model performance: probabilistic accuracy, respect for the ordinal structure, and classification agreement, respectively. While these metrics were computed to validate methodological robustness, they were excluded from this manuscript to maintain focus on the study's primary objectives and key contributions.

### Installation of MPS package and its main functions for categorical multitrait selection

To bridge the gap between theoretical innovation and practical application in plant breeding, we implemented a novel framework for multitrait ordinal selection by integrating Bayesian decision theory with loss functions for multiple ordinal traits, operationalized through the MPS package in R. This approach directly addresses the need for actionable methodologies in GS.

MPS (https://github.com/bjesusvh/MPS) is an R package introduced in our previous work (Villar-Hernández et al. 2024) to face multitrait selection in continuous traits. In this work, we extended its capabilities to facilitate categorical trait selection, incorporating 2 main functions OrdinalPS for single trait selection and MultitraitOPS for categorical multitrait selection. The MPS-R package can be installed by running devtools::install_github(“bjesusvh/MPS”) in the R console. After installation, we can load into the R environment as usual: library(MPS). The user can get help by typing help(OrdinalPS) and help(MultitraitOPS). The inputs and outputs of the functions are described below.

OrdinalPS(Xcand, B, thresholds, target = NULL, method = “kl”)

MultitraitOPS(Xcand, B, thresholds, target = NULL, method = “kl”)

- Xcand is a matrix of predictors for the candidates to be selected, with dimensions n×k, where *n* is the number of candidates and *k* is the number of predictors.
- In the OrdinalPS function, B is a matrix containing regression coefficients, with dimensions M×k, where *N* is the number of MCMC samples after burnIn and *k* is the number of predictors. In the MultitraitOPS function, B is a list of length equal to the number of traits, where each element of B is a matrix of regression coefficients with dimensions M×k, where *M* is the number of MCMC samples and *k* is the number of predictors.
- In the OrdinalPS function, thresholds is a matrix of threshold values from ordinal regression, with dimensions M×t, derived from MCMC samples. Thresholds are the values that define the boundaries between ordinal categories in the outcome variable. They are cut points on the underlying scale of the latent variable that define the level boundaries. In the MultitraitOPS function, thresholds is a list of matrices containing ordinal regression thresholds, with dimensions M×t, derived from MCMC samples.
- In the OrdinalPS function, target is a vector containing probabilities or proportions for the categories within this trait that the breeder wants to achieve in a long-term breeding program. The proportions must sum to 1. Zero entries are not allowed. In the MultitraitOPS function, target is a list of vectors, where each vector corresponds to an ordinal trait. Each vector must contain probabilities or proportions for the categories within that trait.
- In both functions, method is a string specifying the loss function to use. Options are “kl” for Kullback–Leibler, “hellinger,” and “bhattacharyya.” The default is “kl.”

The outputs of the above functions are R-list objects containing, for each candidate line, the posterior expected loss (EL), the ranking, and either the predicted level or the predicted probability for each level. Breeders should select the lines with the lowest loss or the best ranking (1 = best, 2 = second best, and so on).

### Simulation study—scenarios

Simulation tools play a crucial role in optimizing breeding programs and evaluating GS strategies. Therefore, in this paper, we present the results of a simulation approach using loss functions under multitrait categorical traits framework to identify the best potential candidates as parents of the next generation in a recurrent simulation program with 10 cycles. In each selection cycle, an offspring of full siblings was derived from parents randomly selected from the entire population. From each offspring, lines of double haploids were randomly generated, resulting in a total of 2,000 lines in each cycle. To represent historical evolution and induce linkage disequilibrium (LD), 200 generations of random mating were simulated in a population of 2,000 lines, segregating for all loci. The allele frequency was set to 0.5. The simulated genetic component follows Mendelian segregation laws for diploid species. The genome consisted of 5,000 independently segregating sites.

We simulated 3 categorical traits (traits 1, 2 and 3) based on the discretization of 3 quantitative traits. Each categorical trait has 3 categories: level 1 (less desired level), 2, and 3 (most desired level). The quantitative traits were simulated assuming 2 narrow sense heritability values, $h^{2}=0.3$ to mimic a low heritability and $h^{2}=0.6$ to represent a moderate scenario. These values were fixed for all traits. Genetic correlations between quantitative traits were as follows:


$$\begin{array}{rl} & S_{1}=\left[\begin{array}{ccc} 1 & 0.3 & 0.3\\ 0.3 & 1 & 0.2\\ 0.3 & 0.2 & 1 \end{array}\right],\;S_{2}=\left[\begin{array}{ccc} 1 & -0.3 & -0.2\\ -0.3 & 1 & -0.35\\ -0.2 & -0.35 & 1 \end{array}\right],\\ & S_{3}=\left[\begin{array}{ccc} 1 & 0.5 & -0.4\\ 0.5 & 1 & 0.3\\ -0.4 & 0.3 & 1 \end{array}\right] \end{array}$$

where $S_{1}$ reflects 3 positively correlated traits, $S_{2}$ traits have opposing genetic effects, useful for studying antagonistic pleiotropy or trade-offs between traits (such as yield and resistance), and $S_{3}$ represents a mixture of positive and negative correlations, reflecting complex genetic relationships. We tested all combinations of $h^{2}$ and *S*, resulting in 6 scenarios: $E_{1}(S_{1}$ & $h^{2}=0.3)$, $E_{2}(S_{1}$ & $h^{2}=0.6)$, $E_{3}(S_{2}$ & $h^{2}=0.3)$, $E_{4}(S_{2}$ & $h^{2}=0.6)$, $E_{5}(S_{3}$ & $h^{2}=0.3)$, and $E_{6}(S_{3}$ & $h^{2}=0.6)$. True gene effects were performed by randomly sampling gene effects for all segregating sites from a multivariate normal distribution with a mean of 0 and a previously specified variance-covariance to ensure genetic correlation $S_{1}$, $S_{2}$, and $S_{3}$.

The population proportion of each level for each categorical trait at F0 was approximately 33.3%. Subsequently, 70% of these lines were used to train the ordinal regression model using the BGLR-1.1.3 software (Pérez-Rodríguez and de los Campos 2014); for each ordinal trait, we fitted an individual ordinal regression model. Thirty percent of the remaining lines were used as a pool of candidate individuals for selection, with 30% selection pressure. Selection was performed by ranking individuals based on their EL from lowest to highest. The selected lines were then randomly crossed to form the new improved population. This process was repeated 20 times (Monte Carlo replicates) for each of the loss functions proposed in this work: KL divergence, BD, and Hellinger distance. For each ordinal trait, we defined a reference distribution *q* = (0.1, 0.2, 0.7), which means that in the long run, the goal is to reach 10% of lines with level 1, 20% with level 2, and 70% with level 3 for each trait.

The recurrent selection simulation implemented in this study uses unmoderated truncation selection (top 30% based on minimum EL) and random mating among selected individuals. While this setup does not reflect the mating constraints or diversity management strategies used in practical breeding programs, such as control of coancestry, equal parental contributions, or optimal contribution selection, it was intentionally designed to serve as an idealized and unconstrained test bed for comparing divergence-based selection strategies. Our primary goal was to evaluate the ranking behavior and convergence properties of different loss functions under well-defined and replicable genetic architectures, rather than to model the full complexity of breeding pipelines or assess long-term population viability.

Including diversity-preserving constraints, such as limiting relatedness or enforcing balance in parental contributions, would introduce confounding variables that obscure the core objective of this work: to assess how well divergence metrics guide selection toward breeder-defined target distributions. Moreover, many existing studies on optimal contribution strategies are based on additive linear models for continuous traits, whereas our framework addresses the more complex challenge of multitrait ordinal selection with non-Gaussian outcomes. The interaction between loss function-based selection and diversity-preserving mating designs represents an important direction for future work, especially as extensions of our methodology are developed for long-term breeding strategies. In this study, however, we deliberately adopt an unconstrained setting as a form of stress testing, to reveal the intrinsic properties and limitations of each loss function under consistent selection pressure.

Finally, the inclusion of a real data application involving 403 synthetic hexaploid wheat (SHW) lines evaluated for multiple disease resistance traits, where relatedness, inbreeding, and allele frequency dynamics are naturally present, serves as an important complement to the simulation. The alignment of outcomes between simulation and real-world data provides additional support for the robustness and practical value of divergence-based selection under ordinal, multitrait frameworks.

### Real data application of random sampling validation for loss function-based selection

To assess the performance of our selection strategy, we applied a random sampling validation for loss function-based selection using a CIMMYT experimental dataset described below.

A total of 403 SHW lines developed by CIMMYT's Wheat Wide Crosses Program were selected from an initial pool of 1,524 candidates based on resistance to *Fusarium* head blight (character 1), *S. tritici* blotch (character 2), rust (character 3), and other phenological traits. These SHW lines were derived from crosses between 40 durum wheat (DW) parents and 277 *Aegilops tauschii* accessions. The DW parents were used in 1 to 54 crosses, while *A. tauschii* accessions were used in 1 to 7 crosses (Lozano-Ramírez et al. 2022).

Disease screening was conducted in 2018 to 2019 in a greenhouse at CIMMYT, El Batán, Mexico, following a randomized complete block design with 12 replicates for SHW lines and 8 replicates for DW parents. Each experimental unit consisted of 4 plants, with 4 control checks representing different levels of resistance. The experiments were conducted under controlled conditions (22–25 °C day/16–18 °C night, 16-h photoperiod). Genotyping was performed on 10-day-old seedlings using DArTseq™ technology after DNA extraction using a modified CTAB protocol. PstI and HpaII enzymes were used for complexity reduction, and 83 bp fragments were sequenced on the Illumina NovaSeq6000 platform. A total of 19,752 molecular markers were retained for downstream analysis. The traits were scored using an ordinal scale from 1 to 5 where 1 is defined as resistance and 5 as susceptible.

During the validation process, 30% of the lines (121 individuals) in each replicate were randomly sampled and treated as candidates with missing phenotypic values. This setup simulates a realistic breeding scenario where the genetic values of certain lines are unknown and breeders must predict these values in order to identify and select the best potential parents. The remaining 70% (282 lines) were used to train a Bayesian ordinal regression model implemented in the BGLR software (Pérez-Rodríguez and de los Campos 2014). Missing values were predicted as vectors of posterior probabilities via MCMC sampling, which were then used in the R function MultitraitOPS.

Each candidate was ranked using a loss-based selection criterion derived from 1 of 3 divergence measures: KL, Hellinger, or Bhattacharyya. The top 30 individuals (∼25% of the candidates) were selected based on each loss function. As a baseline for comparison, another 30 individuals were randomly selected from the candidate set in each iteration. This allowed us to evaluate the effectiveness of each loss function in identifying favorable phenotypic profiles.

After selection, the frequency of each level per trait was calculated for both the loss function-based and baseline selections. This entire process was repeated 30 times independently for each loss function.

Since level 1 corresponds to the lowest level of disease severity out of a total of 5 (Table 1), our goal was to maximize its frequency among the selected individuals. As a reference, we used the following target distributions: for traits with 4 categories, q=(0.8,0.1,0.05,0.05); for traits with 5 categories, q=(0.8,0.1,0.05,0.025,0.025).

**Table 1.** Distribution of categories for each character in the real dataset, as used in the random sampling validation approach for loss function-based selection.

Category or level	Character 1	Character 2	Character 3
1	59.71%	37.38%	44.74%
2	19.42%	38.59%	42.54%
3	18.45%	21.84%	10.51%
4	2.43%	1.94%	1.71%
5	—	0.024%	0.049%

## Results and discussion

In this section, we present the results of simulation scenarios contrasting the results of the 3 loss functions proposed in this research. After that, we present the results obtained using a random sampling validation approach using a real dataset of disease resistance. Finally, we include one simple example to show users step by step how to use OrdinalMPS (for ordinal single trait parental selection) and MultitraitOPS (for multitrait ordinal parental selection) functions from the MPS-R package.

### Results of simulation scenarios

**Fig. 2.**
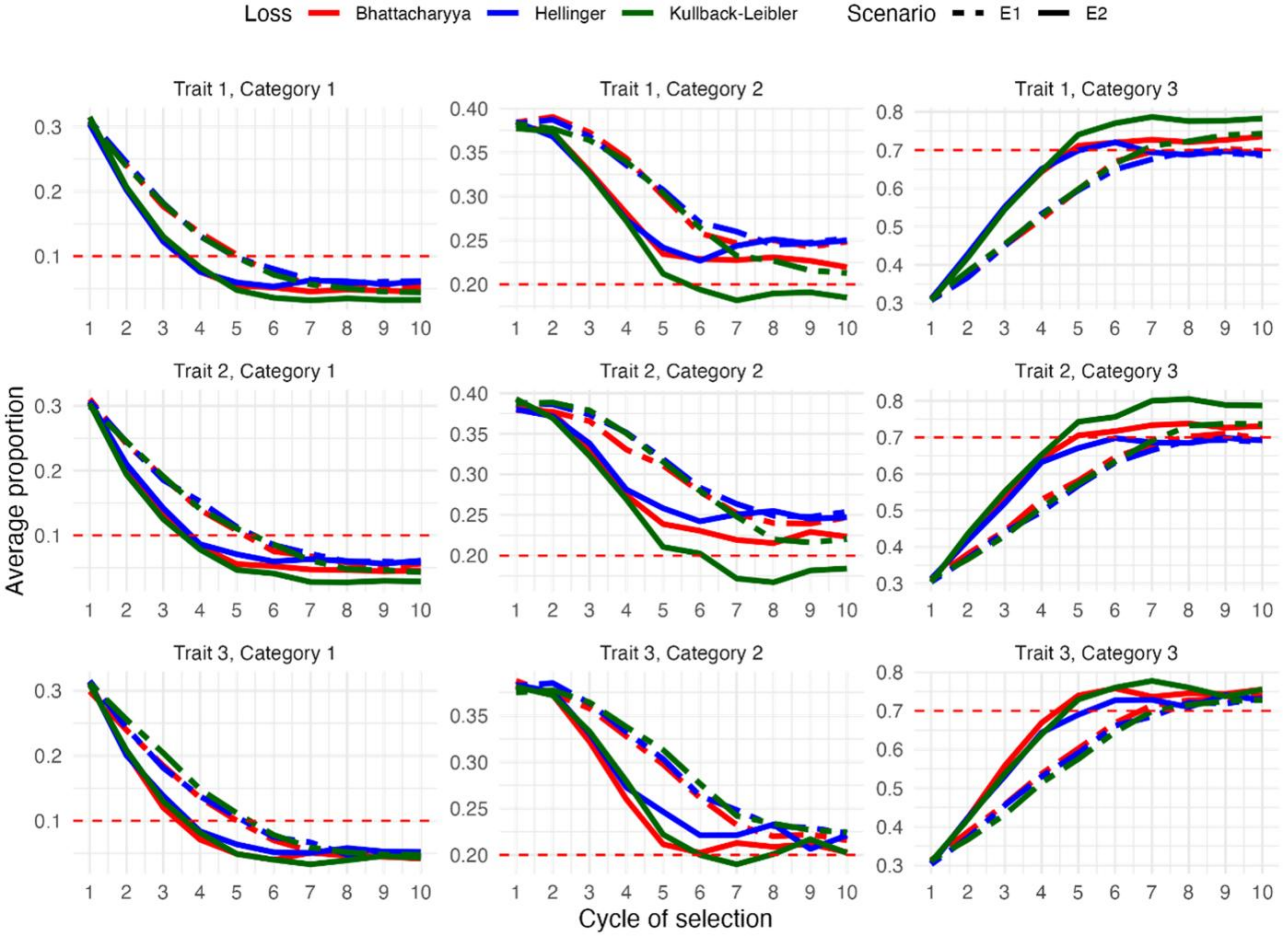
The *y* axis represents the average population frequencies for each level (category 1 A; level 2 B and level 3 C) of ordinal multitraits, evaluated over 10 selection cycles (*x* axis) in 2 experimental scenarios: $E_{1}$ ($S_{1}$ with $h^{2}\;=\;0.3$) and $E_{2}$ ($S_{1}$ with $h^{2}\;=\;0.6$) for all loss functions. Horizontal red dashed lines indicate the target proportions for each level.

The simulation results, based on 20 replicates per cycle under each of the 6 scenarios, $E_{1}(S_{1}$ & $h^{2}=0.3)$, $E_{2}(S_{1}$ & $h^{2}=0.6)$, $E_{3}(S_{2}$ & $h^{2}=0.3)$, $E_{4}(S_{2}$ & $h^{2}=0.6)$, $E_{5}(S_{3}$ & $h^{2}=0.3)$, $E_{6}(S_{3}$ & $h^{2}=0.6)$, were averaged and are presented below. Figure 2 shows the average population proportions for scenarios $E_{1}$ and $E_{2}$, stratified by trait and level. The *x* axis represents the selection cycles, while the *y* axis represents the population proportion. The red dashed horizontal line indicates the target value for each case.

In scenarios where the traits show a positive correlation, the results are in line with theoretical expectations. In the third column, corresponding to the most desirable level (Category 3) of each trait, under scenario $E_{2}$, the target values were achieved from cycle 5 onwards for the 3 loss functions evaluated. On the other hand, in scenario $E_{1}$, this objective was not reached until cycle 7. This difference is attributed to the moderate heritability associated with scenario $E_{2}$ compared to the low heritability of scenario $E_{1}$. From cycle 5 or 7, genetic gains were marginal, with a slight increase when using the KL with respect the other 2 loss functions.

On the other hand, in the least desirable level (category 1) of each trait, shown in the first column of the graph, the selection targets were achieved from cycle 4 in scenario $E_{2}$ and from cycle 5 in scenario $E_{1}$. For level 2, the selection target was reached by cycle 6 for all 3 traits when using the KL function, but only under scenario $E_{2}$ (moderate heritability). Approximations to the target were observed for the remaining combinations of loss functions and heritability levels, with these being more pronounced for scenario $E_{2}$.

In summary, the KL function showed slightly better performance in terms of genetic gains, especially in scenarios with moderate heritability.

**Fig. 3.**
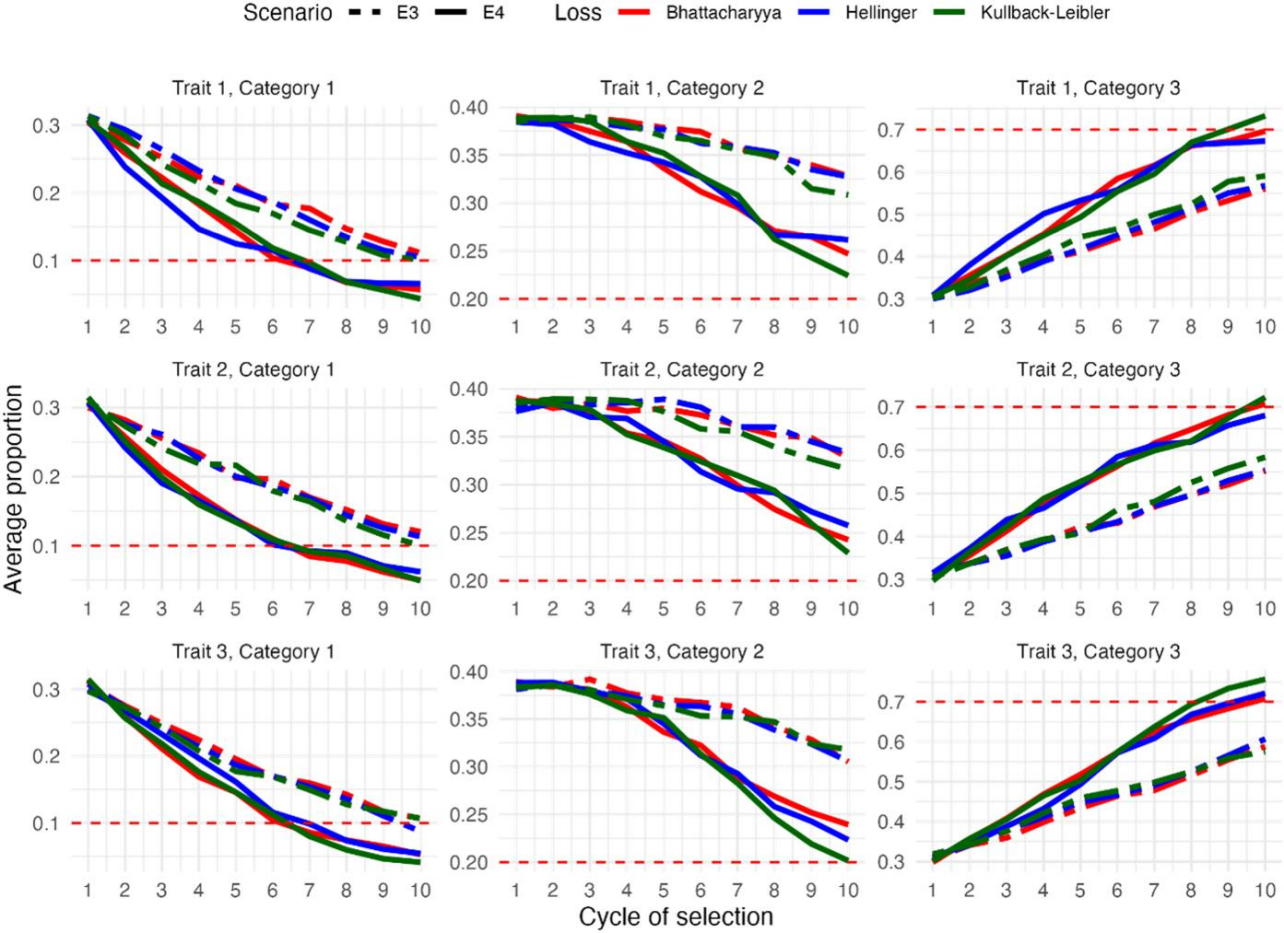
The *y* axis represents the average population frequencies for each level of ordinal multitraits, evaluated over 10 selection cycles (*x* axis) in 2 experimental scenarios: $E_{3}$ ($S_{2}$ with $h^{2}\;=\;0.3$) and $E_{4}$ ($S_{2}$ with $h^{2}\;=\;0.6$) for all loss functions. Horizontal red dashed lines indicate the target proportions for each level.

Figure 3 shows the results obtained in scenarios $E_{3}$ and $E_{4}$, when the correlation matrix $S_{2}$ was used to induce antagonism between traits. As expected in complex selection scenarios, genetic gains were achieved at a slower rate between selection cycles. For level 3 of each trait, representing the most desirable level, the target value was reached between cycles 8, 9, and 10 under the medium heritability scenario ($E_{4}$). However, under the low heritability scenario ($E_{3}$), only about 60% of the target was reached by cycle 10.

In contrast, for level 1 of each trait, corresponding to the least desirable level, the selection target was reached by cycle 7 under the $E_{4}$ scenario, whereas this target was not reached until cycle 10 under the $E_{3}$ scenario. For level 2, the selection objective was partially achieved by the end of the breeding program, with much less satisfactory results in scenario $E_{3}$, where low heritability limited the effectiveness of selection.

Overall, the 3 loss functions evaluated showed similar average performance, with no discernible differences between them. These results highlight the critical influence of heritability and the complexity of antagonistic correlations between traits on the efficiency of genetic selection programs.

**Fig. 4.**
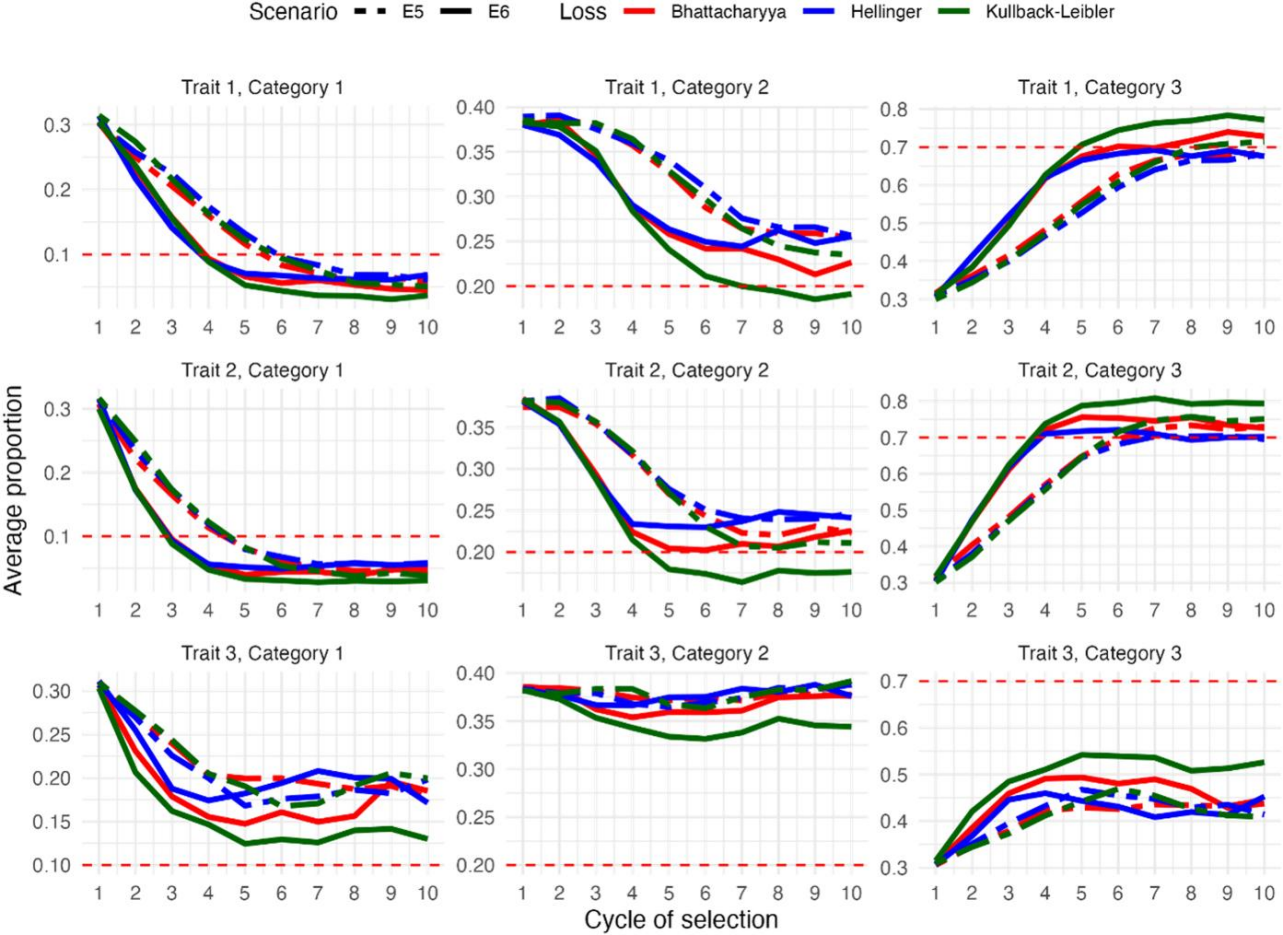
The *y* axis represents the average population frequencies for each level of ordinal multitraits, evaluated over 10 selection cycles (*x* axis) in 2 experimental scenarios: $E_{5}$ ($S_{3}$ with $h^{2}\;=\;0.3$) and $E_{6}$ ($S_{3}$ with $h^{2}\;=\;0.6$) for all loss functions. Horizontal red dashed lines indicate the target proportions for each level.

Lastly, we compare the results of scenarios $E_{5}$ and $E_{6}$ (Fig. 4), both of which used the genetic correlation matrix $S_{3}$ that included both positively and negatively correlated traits. This scenario, which represents complex genetic relationships, produced results that can be considered satisfactory overall.

For example, in level 3 in traits 1 and 2, the selection objectives were achieved as early as cycles 5 and 4 under scenario $E_{6}$, and later, during cycles 8 and 6, under scenario $E_{5}$. In contrast, for level 3 in trait 3, the desired genetic gains were not fully realized. However, an average improvement of 50% relative to baseline was observed, with slightly greater progress achieved using KL divergence.

A similar pattern was observed in level 1 for traits 1 and 2, where the selection goals were achieved by cycles 4, 5, and 6, with scenario $E_{6}$ again showing greater efficiency in achieving the goals. For trait 3 in level 3, the selection objective was partially met, with KL divergence consistently providing better performance.

In level 2, selection objectives were fully met in some cases and partially met in others. For example, for trait 1 under scenario $E_{6}$ (moderate heritability), the KL divergence reached the desired value by cycle 7, followed by the BD, which came close to the target. For scenario $E_{5}$ (low heritability), all loss functions achieved partial success, with comparable results. For trait 2, in both heritability scenarios, the selection objectives were fully or nearly fully met, with the KL divergence again showing superior efficacy. As expected, genetic gains were realized faster under the moderate heritability scenario.

To conclude, for trait 3, all loss functions showed similar performance under both heritability scenarios, with the selection objective remaining unmet. However, it is noteworthy that there was no regression as the population proportions remained stable. The KL divergence slightly outperformed the other metrics, especially under scenario $E_{6}$, although it still did not reach the target.

While previous analyses focused primarily on average outcomes, such as mean population performance or mean loss, a full understanding of the effectiveness of selection strategies requires examining not only central tendencies, but also the variability around them. Because the simulation protocol involved multiple replicates, we obtained empirical distributions for key metrics across selection cycles. This richer structure allows for a more nuanced evaluation that considers both expected performance and associated uncertainty. The following analysis focuses on this second aspect: the variability of outcomes under different scenarios and loss functions, which is critical for assessing the robustness and reliability of GS strategies over time.

To formally test the differences between the loss functions in terms of mean and variance, a regression analysis was performed using a Generalized Additive Model for Location, Scale, and Shape: GAMLSS (Rigby and Stasinopoulos 2005) approach, where “*p*” represents the observed proportion in level 3 of trait *j* = 1, 2, 3 under each of the 6 scenarios:


$$\begin{array}{r} p∼N(\mu ,\;\sigma^{2})\;\;\;\;\\ \mu =\beta_{0}+f_{1}(\mathrm{cycle})+\beta_{1}1_{Hellinger}+\beta_{2}1_{KL}\\ \log(\sigma )=\gamma_{0}+\gamma_{1}1_{Hellinger}+\gamma_{2}1_{KL}\; \end{array}$$

where the mean (*μ*) was modeled by incorporating an intercept term ($\beta_{0}$), a nonlinear term for the cycle modeled using penalized splines $f_{1}(\mathrm{cycle})$, and the effect of the loss functions using the BD as a reference. In this context, $\beta_{1}$ represents the deviation due to the Hellinger distance, while $\beta_{2}$ captures the deviance due to the KL divergence, both relative to the BD. The indicator function 1(⋅) equals 1 if the specified condition is true and 0 otherwise. On the other hand, variance was modeled by the logarithm of the standard deviation, which includes a term for total variability ($\gamma_{0}$) in addition to the effect attributable to the Hellinger distance ($\gamma_{1}$) and the KL divergence ($\gamma_{2}$), both relative to the BD.

The results of this analysis are shown in Table 2. These results indicate that in terms of contribution to the mean, the KL divergence outperforms the BD in 50% of the cases (count the times where KL coefficient has a positive statistically significant value and divide by total of cases, 9/18), while the BD outperforms the KL distance in 5.55% (1/18) of the cases. In the remaining 44.5% of cases, the 2 metrics have identical performance (not statistically coefficients). In contrast, the BD outperforms the Hellinger distance in 50% of the cases, while in the remaining 50%, both measures perform equally well.

**Table 2.** Effects of the Hellinger and the KL with respect to the BD on the mean and variability of the proportion of level 3 in traits 1, 2, and 3 in all scenarios from the simulation study.

Trait category	Loss	Effect on μ	Effect on logσ	Loss	Effect on μ	Effect on logσ
		E1			E2	
T1-C3	Hellinger	−0.006	−0.01	Hellinger	−0.014**	0.126
	KL	0.015***	−0.143	KL	0.028***	0.187
T2-C3	Hellinger	−0.014***	0.052	Hellinger	−0.024***	−0.042
	KL	0.004	0.25*	KL	0.037***	0.203*
T3-C3	Hellinger	−0.011	0.118	Hellinger	−0.021	0.159
	KL	−0.017**	0.014	KL	−0.001	−0.247*
		E3			E4	
T1-C3	Hellinger	0.004	−0.3758***	Hellinger	−0.001	−0.076
	KL	0.02**	−0.1529	KL	−0.001	0.06
T2-C3	Hellinger	0	−0.0904	Hellinger	−0.008	0.205*
	KL	0.014*	0.1303	KL	−0.002	0.328***
T3-C3	Hellinger	0.011	0.1802	Hellinger	0.007	0.072
	KL	0.012	0.0772	KL	0.018*	−0.184*
		E5			E6	
T1-C3	Hellinger	−0.016***	−0.1986*	Hellinger	−0.013*	0.008
	KL	−0.001	0.0033	KL	0.028***	0.26**
T2C3	Hellinger	−0.018***	−0.1709	Hellinger	−0.023***	0.096
	KL	0.003	0.0954	KL	0.034***	0.21*
T3C3	Hellinger	0.008	−0.118	Hellinger	−0.029***	−0.167
	KL	0	−0.2988**	KL	0.045***	−0.013

Significance levels: *P < 0.05; **P < 0.01; ***P < 0.001.

In terms of variability, a negative coefficient indicates reduced uncertainty (less variability of population proportion due to loss functions or more robust loss function). According to this criterion, the BD gives lower uncertainty than KL in 22.2% of the cases, while in 66% of the cases, there is no difference in uncertainty, and in the remaining 22%, KL gives lower uncertainty than Bhattacharyya. Conversely, the Hellinger distance produces lower uncertainty than Bhattacharyya 11% of the time, while 83.5% of the time there is no difference, and only 5.5% of the time does Bhattacharyya produce less uncertainty than Hellinger.

In summary, the results indicate that the KL divergence generally outperforms the BD in increasing the mean proportion of level 3, while the Bhattacharyya and Hellinger distances show comparable performance. In terms of variability, the BD more often shows lower variability compared to the KL distance, while the Hellinger distance rarely outperforms the BD. These results highlight the different effects of divergence measures influenced by heritability and trait correlation structures and emphasize the importance of selecting the most appropriate loss function based on the breeder's specific situation.

### Results from the real data application of random sampling validation approach

**Fig. 5.**
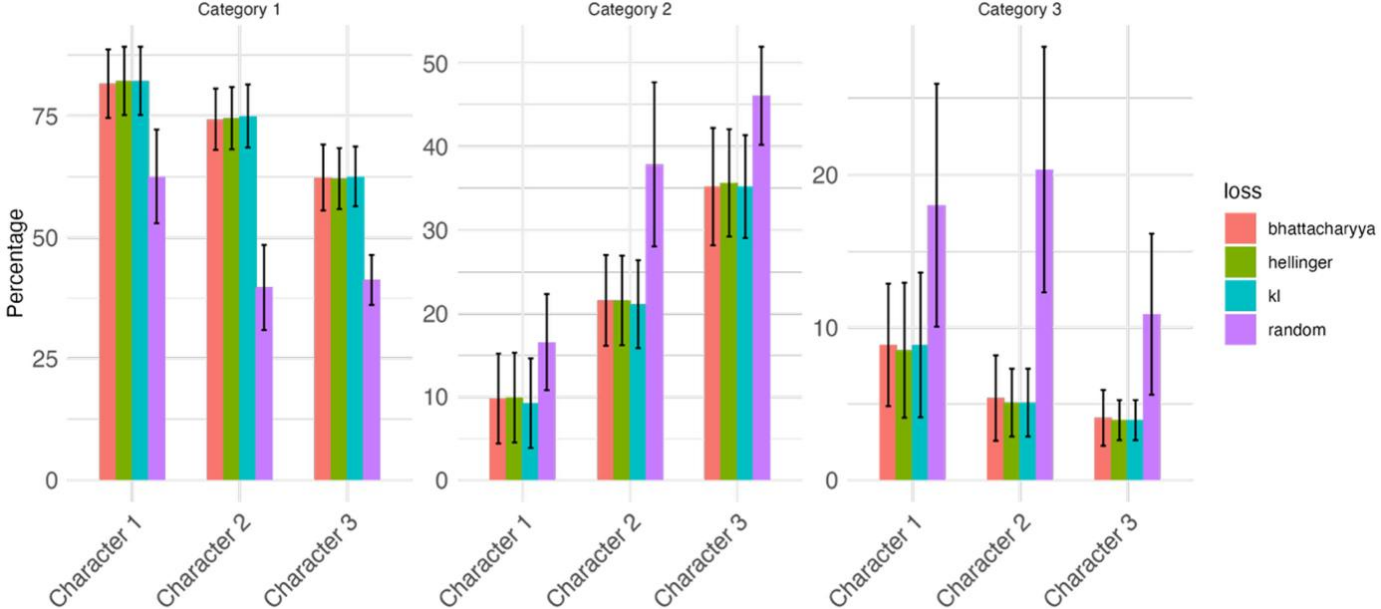
Percentage distribution of each level across characteristics, based on the validation procedure using real data and 3 loss functions, along with a baseline scenario. Bars represent average values, and error bars indicate the standard deviation obtained from 30 repetitions of the random sampling validation for loss function-based selection.

The results of the 30 replicates from the real data application of random sampling approach are shown in Fig. 5. The bars show the average frequency of each level by trait, with standard deviations shown as error bars. All 3 loss functions showed comparable performance. For character 1, up to 82% of the selected lines belonged to level 1, closely matching the reference target. For character 2 and 3, the average frequency of level 1 was approximately 75% and 62%, respectively.

Notably, none of the loss-based selections included individuals from the least desirable categories (4 and 5), and thus, the results are presented up to level 3. These results show that loss-based selection consistently outperforms random selection, particularly in suppressing the frequency of less desirable categories. This suggests that the integration of loss functions into recurrent selection cycles could enable breeding programs to achieve target phenotypic distributions across traits over time. This is in line with results obtained by simulation scenarios presented in this paper under different conditions of heritability and correlation between traits.

### Summary of results

#### Simulation study

To evaluate the performance of the proposed divergence-based selection approach, we conducted a comprehensive simulation study under 6 scenarios combining different heritability levels (ℎ² = 0.2 and ℎ² = 0.5) and genetic correlation structures (positive, antagonistic, and mixed correlations). Each scenario was run over 10 recurrent selection cycles, and the selection process targeted 3 ordinal traits, each with 3 categories.

Across all scenarios, the KL divergence consistently demonstrated superior performance, particularly under moderate heritability. In scenarios with positive genetic correlations, the desired target distribution for the most favorable level (category 3) was reached within 5 to 7 selection cycles, with KL achieving the target earlier and more consistently than the Bhattacharyya and Hellinger distances. The least desirable level (category 1) also declined steadily across cycles, aligning with selection objectives.

In scenarios involving antagonistic correlations, genetic gains were slower and less pronounced. The KL divergence again performed slightly better, though all 3 loss functions showed comparable behavior by the final cycles. When correlations were mixed (both positive and negative), performance varied by trait, with KL achieving better alignment with target distributions in traits 1 and 2, while none of the methods fully met the target for trait 3, although the KL divergence still yielded the highest improvement.

A statistical comparison using GAMLSS models confirmed these trends: KL divergence produced a statistically significant increase in the mean proportion of level 3 in 50% of the cases, while Bhattacharyya did so in only 5.5%. Regarding variability, Bhattacharyya yielded lower uncertainty in a minority of cases, while KL occasionally reduced variability. Hellinger did not show significant advantages over either method.

#### Application to real data

To validate the method in a practical setting, we applied the 3 divergence functions to a dataset comprising 403 SHW lines evaluated for resistance to 3 diseases. Each trait was scored on an ordinal scale from 1 (resistant) to 5 (susceptible), and the genomic dataset included 19,752 DArTseq markers.

In each of 30 replicates, 30% of the lines were randomly masked as candidates, and ordinal regression models were trained on the remaining 70%. Candidates were ranked using KL, Bhattacharyya, and Hellinger divergence, and the top 30 lines were selected based on minimum EL. A baseline of 30 randomly selected candidates was included for comparison.

The results showed that all 3 divergence-based methods substantially increased the proportion of resistant lines (category 1): character 1 reached ∼82%, character 2 ∼75%, and character 3 ∼62%. In contrast, the baseline random selection had lower frequencies of desirable categories and higher frequencies of less desirable ones. Importantly, no individuals in categories 4 or 5 were selected using the loss-based approaches, confirming their effectiveness in avoiding susceptible lines.

Overall, these findings confirm that the proposed divergence-based selection framework—particularly when using the KL divergence—provides a robust, flexible, and effective method for optimizing multitrait ordinal selection in both simulated and real-world plant breeding contexts.

#### An example of how to use MultitraitOPS and OrdinalPS

The goal of this final section is to provide practitioners with a step-by-step guide to using the MPS-R package for ordinal selection in both single-trait and multitrait contexts. First, the necessary libraries are loaded: BGLR for fitting ordinal regression models and MPS for calculating the EL of each candidate line for selection. The dataset used in this example (included in the MPS-R package) is also loaded, consisting of 350 lines. For each line, there is information on 8,281 molecular markers of the SNP type (matrix X), together with phenotypic data for 2 categorical traits (matrix *Y*). Each trait has 4 categories (labeled 1, 2, 3, 4). A seed number is then set to ensure the reproducibility of this example. Next, 50 lines are randomly selected as the candidate set for selection. In real cases, the *Y* values for candidate lines may be completely unknown for all lines or partially missing for some lines or specific traits. Other scenarios may arise in practical situations; for example, a breeder might evaluate the same lines for a single ordinal trait across multiple environments. In such cases, the decision-making objective would shift from selecting the best lines across traits to identifying the best lines across environments. Additionally, breeders may need to evaluate the same lines across multiple environments for multiple traits, further increasing the complexity of the selection process.

Several parameters are then defined for BGLR, including the number of iterations, the burn-in period and the thinning interval for the MCMC samples obtained. The reader is referred to the official documentation of the BGLR package for further details. The last line of chunk 1 checks whether an “out” folder already exists in the working directory; if it does not, the script creates it to store the regression model outputs used as inputs for the MPS package.

##### Chunk 1

library(BGLR); library(MPS)

setwd(“Put you directory here”)

load(ordinalData)

set.seed(63)

id_candidates <- sample(x = 1:nrow(Y), size = 50, replace = FALSE)

YNA <- Y; YNA[id_candidates, ] <- NA

Xcandidates <- X[id_candidates,]

no_iter <- 50e3; no_burn <- 20e3; thin <- 5

id_samples <- which(seq(1, no_iter, thin) > no_burn)

if (!dir.exists(“out”)) {dir.create(“out”)}

Chunk 2 specifies the Bayesian Ridge Regression (BRR) model, which applies a quadratic penalty to the SNP coefficients. Alternatively, the Bayesian Lasso (BL) model can be selected. The saveEffects = TRUE argument instructs BGLR to store the MCMC realizations of the effects for each predictor in X. Two separate models are then trained, one for each trait of interest. It is important to note that if there are more than 2 categorical features, the procedure remains the same, i.e. a separate model must be trained for each feature under selection.

##### Chunk 2

ETA <- list(list(X = X, model='BRR', saveEffects = TRUE))

fmO1 <- BGLR(YNA[,1], response_type = ‘ordinal’,

      ETA = ETA, nIter = no_iter, burnIn = no_burn,

      thin = thin, verbose = FALSE, saveAt = “./out/O1”)

fmO2 <- BGLR(YNA[,2], response_type = ‘ordinal’,

      ETA = ETA, nIter = no_iter, burnIn = no_burn,

      thin = thin, verbose = FALSE, saveAt = “./out/O2”)

Chunk 3 loads the MCMC regression coefficients associated with each SNP. Similarly, the MCMC thresholds obtained from the ordinal regression model are also loaded. These 2 inputs are then passed to the MPS package to proceed with the EL calculation.

##### Chunk 3

# Read MCMC samples from ordinal model 1

beta_1 <- readBinMat(paste0(file.path(“./out/O1ETA_1_b.bin”)))         

thrO1 <- as.matrix(read.table(“./out/O1thresholds.dat”)[id_samples,])

# Read MCMC samples from ordinal model 2

beta_2 <- readBinMat(paste0(file.path(“./out/O2ETA_1_b.bin”)))

thrO2 <- as.matrix(read.table(“./out/O2thresholds.dat”)[id_samples,])

Finally, in chunk 3, the MultitraitOPS function is run to calculate the EL for each candidate line to be selected. We use the “bhattacharyya” loss function as an example. However, users can replace this function with the available alternatives “kl” or “hellinger” by specifying the desired option in the method argument. Note that in target argument, we specified a list with 2 vectors. Note that in the target argument, we specify a list with 2 vectors: the first entry represents the reference/target distribution for trait 1, and the second entry represents the reference distribution for trait 2.

The output of the MultitraitOPS function includes the EL for each candidate line, its ranking based on loss (from lowest to highest, where ranking = 1 is the best and ranking = 50 is the worst), and the predicted level for each trait.

##### Chunk 3

B <- list(beta_1, beta_2)

thresholds <- list(thrO1, thrO2)

target <- list(c(0.1, 0.2, 0.2, 0.5), c(0.1, 0.2, 0.2, 0.5))

results <- MultitraitOPS(Xcand = Xcandidates,

      B = B, thresholds = thresholds,

      target = target, method = “bhattacharyya”)

str(results)

List of 3

 $ loss   : num [1:50] 0.382 1.224 0.32 1.105 0.68 …

 $ ranking : num [1:50] 8 48 3 45 40 37 33 5 39 22 …

 $ Cat_Predicted:List of 2

  ..$ Trait 1: int [1:50] 4 1 4 1 2 2 2 4 1 2 …

  ..$ Trait 2: int [1:50] 4 1 4 1 1 2 2 4 2 2 …

In the previous example, simultaneous selection across multiple ordinal traits was considered. However, users may also focus on a single trait. For this purpose, the OrdinalPS function has been designed. Suppose our interest is solely in trait 1; in chunk 4, only the necessary arguments for selection in trait 1 are provided. In this scenario, the output of the OrdinalPS function includes, for each of the 50 candidate lines, the EL, the ranking based on loss (where lower values indicate better candidates), and the predicted probability for each level.

#### Chunk 4

target1 <- c(0.1, 0.2, 0.2, 0.5)

results_t1 <- OrdinalPS(Xcandidates, beta_1, thrO1, target1, method = “bhattacharyya”)

str(results_t1)

List of 4

 $ method : chr “bhattacharyya”

 $ loss : num [1:50] 0.207 0.633 0.175 0.574 0.356 …

 $ ranking: num [1:50] 7 48 3 45 38 37 34 5 40 21 …

 $ yHat  :'data.frame':  50 obs. of 4 variables:

  ..$ Prob_Cat_1: num [1:50] 0.1022 0.6276 0.0634 0.5777 0.3041 …

  ..$ Prob_Cat_2: num [1:50] 0.252 0.251 0.227 0.297 0.314 …

  ..$ Prob_Cat_3: num [1:50] 0.2648 0.0847 0.2803 0.0942 0.1986 …

  ..$ Prob_Cat_4: num [1:50] 0.3811 0.0366 0.4297 0.0309 0.1836 …

 - attr(*, “class”)= chr “MPS”

### Methodological considerations

While GBLUP remains a standard tool for GS of continuous traits, its assumptions of normality and homoscedasticity make it unsuitable for ordinal data. Ordinal traits are inherently discrete, ordered, and often exhibit nonlinear biological transitions, properties fundamentally incompatible with GBLUP's continuous framework. Treating them as such violates statistical assumptions, yielding biased estimates and biologically uninterpretable predictions (Agresti 2010; Montesinos-López et al. 2015; Song et al. 2024).

The Bayesian threshold model addresses these limitations by explicitly modeling ordinal structure through latent liabilities and threshold parameters. This approach not only respects the data-generating process but also provides full posterior predictive distributions. These distributions enable direct comparison with breeder-defined targets via divergence metrics (KL, Bhattacharyya, Hellinger), creating a goal-oriented selection framework impossible under GBLUP.

We emphasize that GBLUP's utility for continuous traits does not extend to ordinal contexts. Our results demonstrate that proper ordinal modeling yields more robust and interpretable selections. While GBLUP serves as a valuable benchmark for quantitative traits, its misapplication to ordinal data risks misleading conclusions. The decision-theoretic approach presented here bridges this methodological gap, aligning statistical rigor with biological reality.

Our simulation study used independently segregating loci to evaluate divergence-based selection metrics (KL, Bhattacharyya, Hellinger) for ordinal traits, intentionally omitting LD structures. This simplified design was justified for 3 reasons. First, the study focuses on comparing loss functions' ability to align genotype distributions with target probabilities, not genomic prediction accuracy or marker-QTL persistence. Second, with known simulated marker effects and no QTL inference needed, LD becomes irrelevant to the decision-theoretic framework. Third, independent loci provide a controlled benchmark to isolate loss-function performance.

The simulation tested 6 genetic architectures under recurrent truncation selection (top 30% selected) using random mating without constraints. This design stress-tested convergence dynamics under maximum selection pressure. While idealized, this approach clarifies how divergence metrics drive selection when freed from confounding factors like LD or mating restrictions.

We validated the approach using 403 synthetic wheat lines (20K SNPs) with natural LD and ordinal disease resistance traits. Real-world results confirmed the simulation findings, demonstrating robustness under genomic complexity. This 2-part methodology provides both controlled mechanistic insights and practical validation.

A key distinction is that our framework evaluates probabilistic alignment with breeding targets rather than marker-trait associations. Since divergence metrics operate on posterior distributions of ordinal categories, physical genome structures do not affect their theoretical comparison. Thus, while LD matters for genomic prediction studies, its exclusion here maintains focus on our core innovation: a decision-theoretic system for ordinal trait selection. Future work could incorporate mating constraints, but this study prioritized isolating loss-function effects to establish foundational principles.

## Conclusions

This work represents a natural extension of our previous studies (Villar-Hernández et al. 2018, 2024), where divergence-based selection was introduced for continuous traits. Here, we broaden the scope to ordinal traits, which are prevalent in disease resistance and other categorical breeding targets, and develop a principled Bayesian framework for multitrait ordinal selection. This work presents a significant advancement in GS methodologies by introducing a Bayesian decision-theoretic framework specifically tailored for multitrait ordinal data. By employing information-theoretic divergence metrics, our method allows breeders to define and directly optimize selection goals as target probability distributions, moving beyond traditional regression-based approaches. Simulation studies across diverse genetic architectures confirmed the robustness and predictive strength of this approach, with KL divergence frequently emerging as the most effective loss function under moderate heritability. The Bhattacharyya and Hellinger distances also proved valuable, particularly in scenarios with complex trait correlations. Importantly, real-world validation using SHW data demonstrated that the proposed strategy outperforms random selection, enabling meaningful improvements in desirable trait frequencies. The integration of these tools into the MPS-R package ensures accessibility and reproducibility, equipping breeders with a powerful tool to enhance genetic gains in ordinal trait selection. Future research should explore extending this framework to accommodate genotype-by-environment interactions and incorporate nonlinear trait dependencies, further enhancing its utility in modern plant breeding.

## Data Availability

Repository containing supplementary materials, data, and code for the paper Bayesian Divergence-Based Approach for Genomic Multitrait Ordinal Selection (G3 Genes|Genomes|Genetics) can be accessed at https://github.com/bjesusvh/Bayesian-Divergence-Based-Approach-for-Genomic-Multitrait-Ordinal-Selection/tree/main.
